# Glioma-neuron interactions: insights from neural plasticity

**DOI:** 10.3389/fonc.2025.1661897

**Published:** 2025-09-11

**Authors:** Jingyu Feng, Jun Yang

**Affiliations:** ^1^ Department of Neurosurgery, Peking University Third Hospital, Peking University, Beijing, China; ^2^ Center for Precision Neurosurgery and Oncology of Peking University Health Science Center, Peking University, Beijing, China

**Keywords:** glioma, neural mechanisms, neuroplasticity, neural networks, migration, glioma stem cell, tumorigenesis, tumor microenvironment

## Abstract

The development of gliomas is linked to neuroplasticity. Neurons, which are largely nonregenerative in adulthood, rely on axons and synapses to rebuild the neural network in response to experience and injury. Neural stem cells and immune cells coordinate “creation” (e.g., neurogenesis) and “clearance” (e.g., synaptic pruning), guided by signals from neural circuits. This review summarizes neuroplasticity mechanisms and explores their connection to gliomas, revealing that glioma cells hijack neural network derived signals to promote growth, migration, and stem-like properties, while simultaneously disrupting normal neural conduction. Similar to oligodendrocyte precursor cells (OPCs), gliomas exploit neural network regulation but are prone to uncontrolled proliferation. Moreover, glioma induced neural hyperexcitability disrupts circuit homeostasis, creating a permissive microenvironment for glioma progression. Consequently, neuroplasticity will contribute to the study of glioma related mechanisms and the development of more targeted strategies for prevention and control.

## Graphical Abstract

A schematic that visually defines the structure of this review.

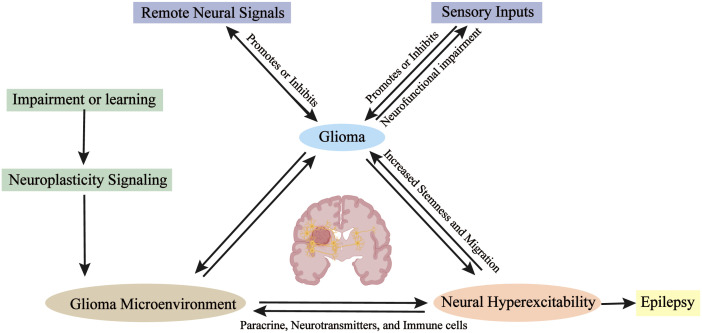

## 1 Introduction

Gliomas, which originate from central nervous system (CNS) cells and account for 75% of malignant primary brain tumors in adults, are the most common type of primary brain tumor (1–5). Classically, tumor cell proliferation was regarded as an “autonomous” process driven by genetic defects, with neural signaling interactions considered secondary (6). However, recent evidence indicates glioma cells are active participants, expressing neuron-like ion channels and neurotransmitter receptors to decode neural signals and regulate invasion, metabolism, and drug resistance (7, 8).

In the normal brain, neurons form a complex signaling network through electrical activity and neurotransmitter release (e.g., glutamate, γ-aminobutyric acid (GABA)) to regulate cognition and movement, a process termed neural plasticity (9, 10). Histopathologic and lineage analyses confirm that neural stem cells (NSCs), glial progenitors (e.g., OPCs), and astrocytes are potential origins of gliomas (11–13). These cells are all involved in regulating nervous system plasticity in the brain (14). It is widely recognized that cancer arises from the dysregulation of homeostatic mechanisms governing tissue repair and stem cell self-renewal (15). In the adult brain, NSCs and glial progenitor cells exhibit characteristics associated with central nervous system cancers, including a strong proliferative potential and diversity (16). Meanwhile, NSCs are regulated by the same cellular pathways that are active in brain tumors, such as the Notch, Wnt, and NF-κB signaling pathways (17–19). In the stem cells of the adult brain, OPCs constitute a major proliferative population, uniformly distributed throughout the adult rodent brain (20, 21). Numerous studies have shown that OPC or earlier pre-OPC cells are present in various forms of gliomas (22, 23). Dysregulation of myelin plasticity promotes glioma cell proliferation in primary brain cancer (24). Synaptosomal-associated protein 25 (SNAP25), a synaptic plasticity protein, is significantly correlated with the progression of glioma (25, 26). In summary, neuroplasticity is closely linked to the initiation and progression of gliomas, particularly in myelin plasticity. Aberrant plastic repair mechanisms may drive the development of gliomas, while post-glioma repair processes can further promote glioma progression.

**Figure 1:**
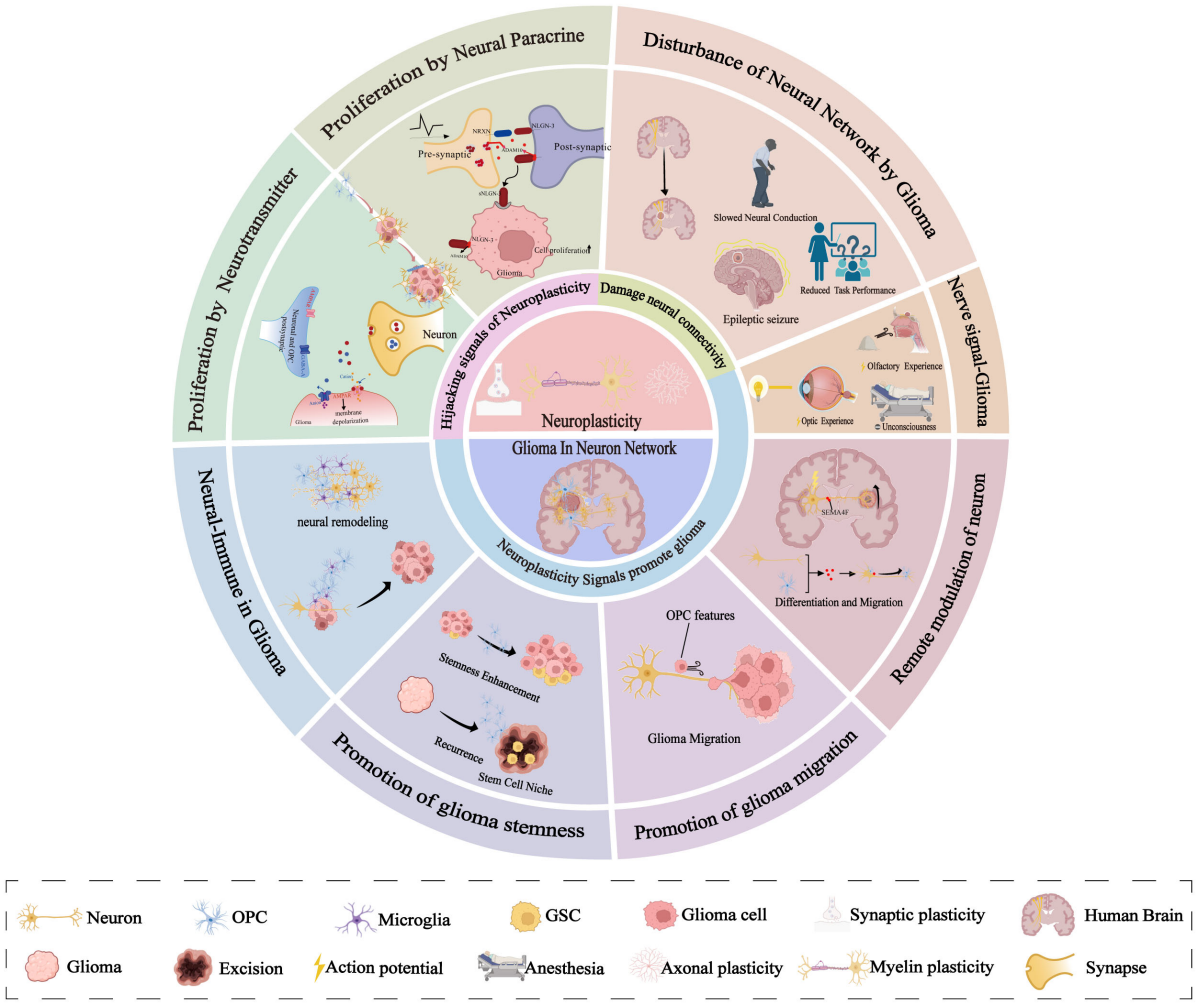
Specific aspects of glioma-neuron interactions and neural plasticity. Neural plasticity governs myelin, axon, and synaptic remodeling via neuronal signaling, with gliomas preferentially arising in highly plastic regions. Gliomas hijack neural stem cell repair mechanisms—forming synapses to receive neurotransmitters and paracrine factors—while neural inputs (e.g., visual/olfactory stimuli, anesthesia) significantly impact glioma growth. Bidirectional glioma-neuron communication drives epileptogenesis and impairs functions. Distant neurons participate via neural networks. Neuronal signals enhance glioma cell migration and the acquisition of stem-like properties. Neurons also indirectly regulate gliomas through immune cell crosstalk.

This review summarizes current knowledge on glioma-neuron interactions from the perspective of neuroplasticity, dissecting the intricate mechanisms and structural alterations underlying neuroplasticity. Previous studies have discussed the interrelationship between myelin plasticity and glioma-neuron interactions, proposing that gliomas hijack myelin growth signals to promote self-proliferation (27, 28). Based on these findings, we analyze the relationship between normal neuroplasticity and abnormal glioma behavior in terms of proliferation, migration, stem-like properties, and immune interactions. We further explore the impacts of local neural signals, remote neural signals, and external signals on gliomas (**Figure 1**). We found that glioma-neuron interactions closely resemble the mechanisms of neuroplasticity, but disrupt the homeostatic balance inherent to normal neuroplasticity. The objective of this review is to delineate the correlations between neuroplasticity and glioma-neuron interactions, offering promising future directions for research.

## 2 Neuroplasticity

Neuroplasticity refers to the brain’s capacity to reorganize its structure, function, or connectivity in response to intrinsic or extrinsic stimuli, a process that elicits both functional and morphological alterations. This dynamic process allows us to adapt to different environments and plays an important role in learning, memory, and injury recovery (29). It is well known that neurons in the adult brain are not regenerative upon death (30). Consequently, the remodeling following injury and learning primarily relies on the regrowth and reinnervation of axons (31). Axonal growth forms or strengthens more synaptic connections. Synaptic connections are highly plastic, with the number and strength of synapses changing significantly during development or in response to training (32).

**Figure 2:**
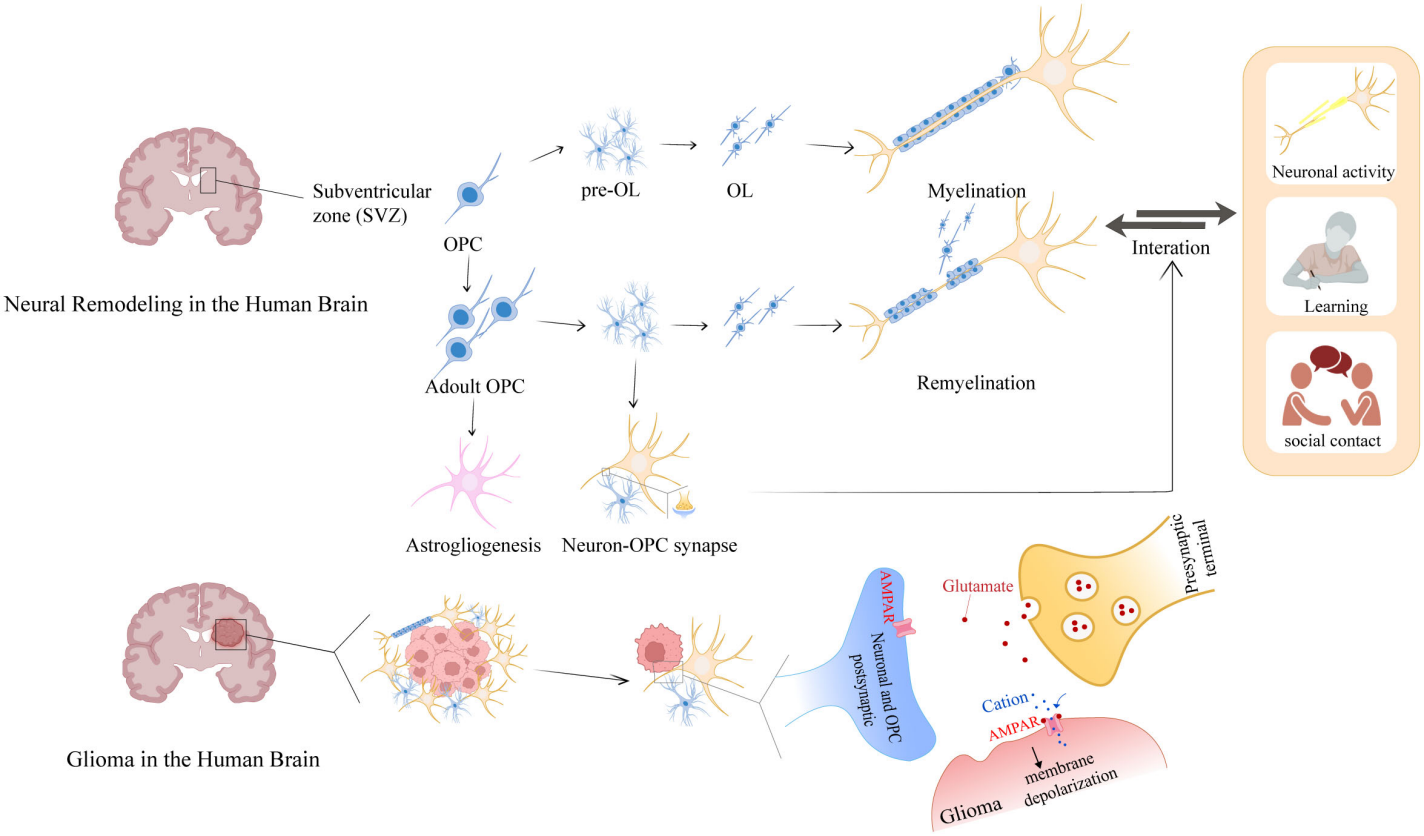
Glioma-neuron interactions of myelin plasticity and glioma intervention. During development, the SVZ serves as the primary source of OPCs. In the adult brain, OPCs that are distributed and reserved throughout the brain establish synaptic connections with neurons to receive repair and remodeling signals. These signals drive OPC proliferation and differentiation into mature OLs, which myelinate axons to facilitate neural network formation. Notably, glioma harbors OPC-like glioma cells that usurp this neuroregulatory pathway: these cells aberrantly repurpose neuronal-derived signals—originally dedicated to myelin repair—for autonomous growth, thereby illuminating a pivotal crosstalk between myelin plasticity and oncogenic mechanisms.

Glia cells act as active regulators of neuroplasticity through their interactions with neurons and exhibit structural plasticity during learning (33, 34). The glia-neuronal crosstalk differs in physiological conditions and various brain disorders (35). Axonal growth relies on the regeneration of myelin sheaths. During the development of the brain, Oligodendrocytes (OLs) are the myelinating cells of the CNS that are generated from OPCs (36), which proliferate and differentiate during embryonic development. OPCs originate in the subventricular zone (SVZ) (37). However, to promote brain repair, OPCs are typically distributed throughout the gray and white matter, where they exhibit strong proliferative and migratory capabilities, as well as the constant capacity for surveillance (20, 38). During the development, myelin sheaths are “optimally distributed” throughout the nervous system. After developmental maturation, OPCs generate OLs involved in adaptive myelination (39). Neuronal activity, learning, and socialization influence myelin formation, this dynamic change in turn regulates signaling in neural circuits and is associated with emotional and cognitive functions, known as “myelin adaptation” (40) (**Figure 2**). Consequently, neuroplasticity can be summarized as three interrelated aspects of axonal plasticity myelin plasticity, and synaptic plasticity, which together form the basis of brain adaptation and plasticity.

## 3 Neural signals influencing glioma growth

Studies have shown that neuronal activity can drive glioma progression via synaptic connections (7, 8), with early neuronal activity found to promote OPC proliferation and differentiation (41). A mouse model in which general anesthesia was used to reduce neuronal activity has demonstrated that low neural signals inhibited the growth and invasion of patient-derived glioblastoma (7). In normal physiological conditions, neural signals from external sensory stimuli can directly impact glioma development, and manipulating olfactory receptor neuron activity influences glioma progression (42). Additionally, stimulation of optic nerve activity promotes optic nerve glioma growth, while reducing visual input inhibits tumor formation and maintenance (43). Surprisingly, radiotherapy—a common treatment modality—accelerates tumor growth by enhancing neuronal activity (44). Collectively, these results indicate that neural signals promote glioma proliferation and differentiation, with such signals being moderately associated with learning and remodeling of the nervous system.

### 3.1 Synaptic transmission

Myelin plasticity homeostasis is important for the prevention of gliomas. The structural basis of myelin adaptation lies in OPCs’ ability to form true synapses with glutamatergic and GABAergic neurons, suggesting neuronal electrical activity regulates OPC proliferation and differentiation (45). In OPCs, GABA positively stimulates signaling cascades, which promote myelination as well as neural recovery (46, 47). Recent studies have further revealed that the OPC can receive inputs from multiple brain regions, illustrating that OPCs have strikingly comprehensive synaptic access to brain-wide projection networks (48). OPC postsynaptic molecules gradually lose the ability to be modulated by neurons during differentiation. As the unique glial cell to forms synapses with neurons, OPCs can accurately predict the location of future myelin production (49).

In recent years, Michelle Monje has illustrated the formation of structural synapses between glioma cells and neurons in the tumor microenvironment through electrophysiological and ultrastructural observations in two works from 2019 (7, 8), which opens up new directions for researchers. Interestingly, it has been revealed through single-cell transcriptomics that glioma cells forming synaptic structures predominantly belong to an OPC-like subpopulation (8). Spontaneous glutamatergic postsynaptic currents are present in such cells. It has been demonstrated that neuronal action potentials induce spontaneous inward currents (SIC) in GB, thereby promoting cancer development (7).

**Figure 3:**
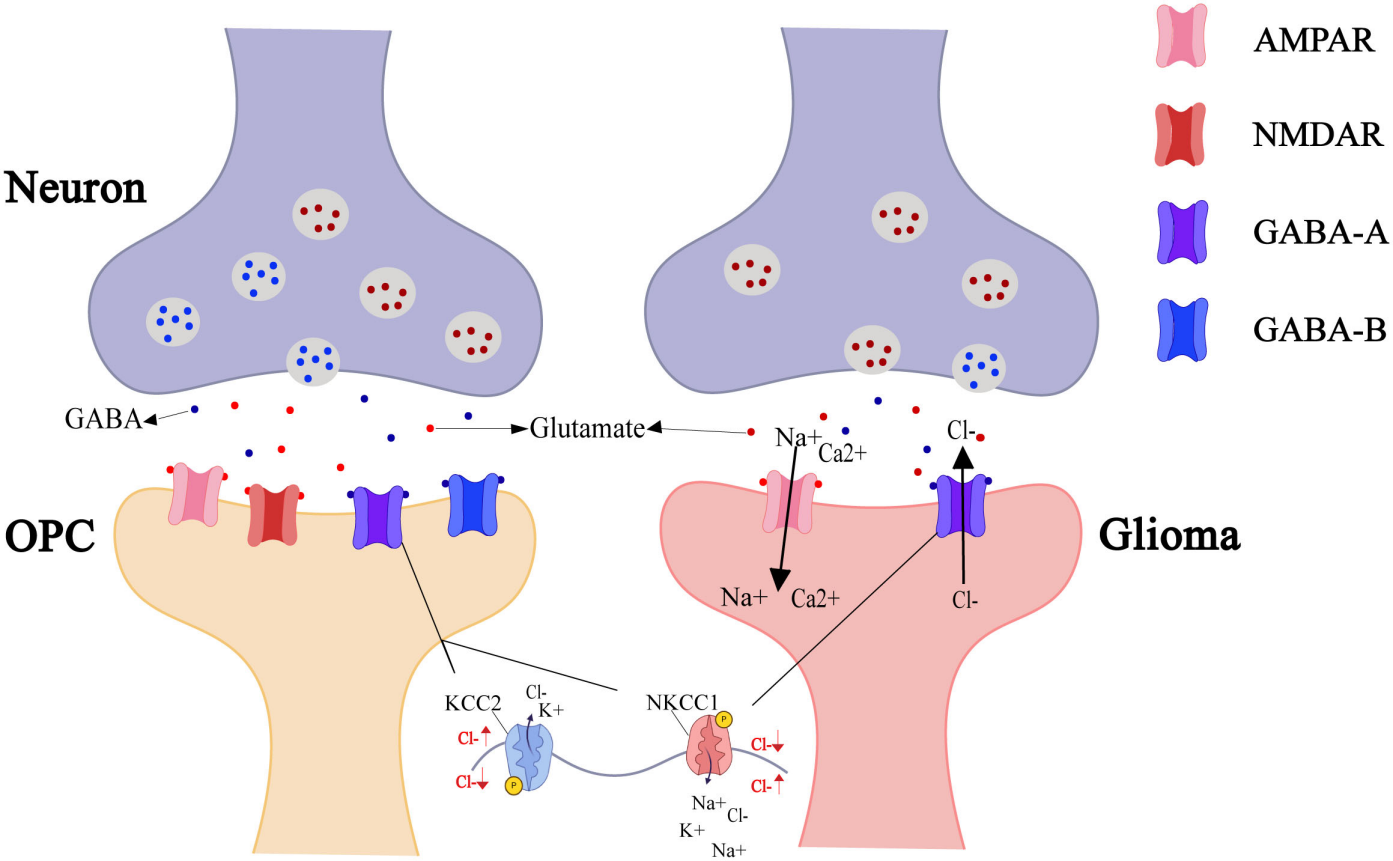
Neuro-OPC vs. neuro-glioma synaptic transmission. As the only glial cells forming synapses with neurons, OPCs undergo neural remodeling and repair regulated by neuronal-released neurotransmitters (e.g., GABA, glutamate). OPC postsynaptic membranes express AMPAR, NMDAR, GABA-A, and GABA-B receptors to integrate excitatory/inhibitory signals from the neurons. In contrast, certain glioma cells also express AMPAR and GABA-A receptors to promote self-growth. AMPAR activation opens Na^+^/Ca²^+^ channels, with cation influx inducing membrane depolarization. GABA-A activation opens Cl^-^ channels, causing efflux of intracellularly accumulated Cl^-^ (due to NKCC1 transporter activity) and inducing depolarization.

Neuron-glioma signal transmission occurs via calcium permeable AMPA receptors. Glutamate released from presynaptic membranes triggers depolarization by activating AMPARs on glioma cell membranes, with receptor inhibitors significantly impeding synaptic communication (7). Similarly, neuronal activity that promotes OPC myelination also involves AMPA glutamate receptors (50). However, in addition to glutamatergic synapses, GABAergic synapses have recently been discovered between gliomas and neurons, with both types able to coexist on a single glioma cell (51). Similar to OPCs in the early developmental stage, the Na-K-2Cl cotransporter (NKCC1) elevates Cl− levels in glioma cells (52), which tends to an efflux of Cl− upon activation of GABAARs. Therefore, GABAergic neuron-to-OPC and GABAergic neuron-to-glioma cell synapses cause depolarization (53). Notably, mature OPCs receive GABA-A-mediated inhibition through upregulated K^+^-Cl^-^ cotransporter 2 (KCC2), while early developmental OPCs respond to promotive signals (47), but gliomas show only promotive effects (**Figure 3**).

Glioma cells exhibit cellular properties similar to those of OPCs, suggesting that interactions between these cancer cells and neurons may be informed by the known neuronal regulation of their putative cellular origins (27).

### 3.2 Neural paracrine NLGN-3

**Figure 4:**
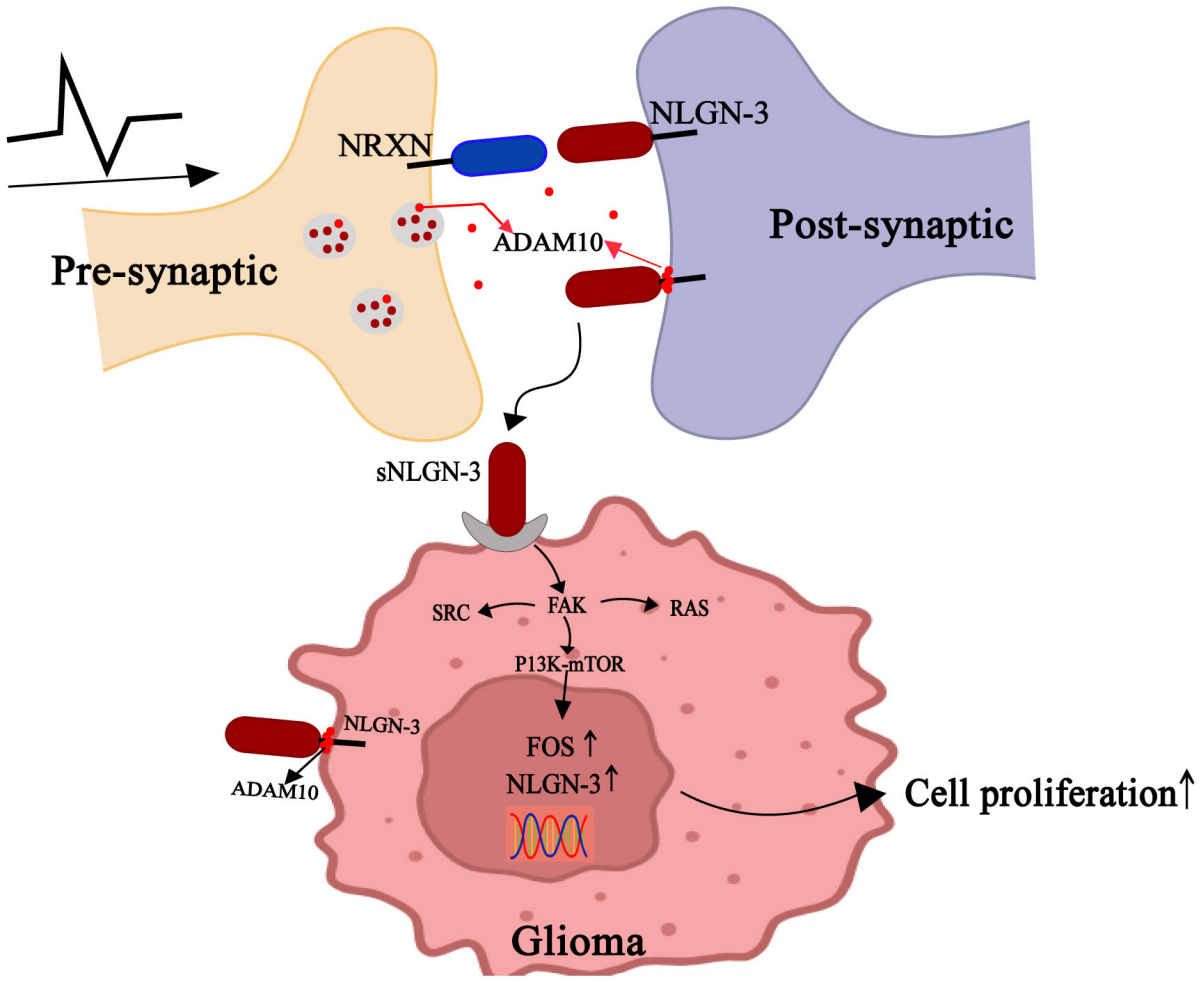
Gliomas exploit paracrine signals of neural plasticity to promote self-development. Neurons release ADAM10 from synaptic vesicles in an activity-dependent manner, which cleaves NLGN3 on neuronal or OPC membranes to shed s-NLGN3. Glioma cells competitively combine s-NLGN3 to activate multiple oncogenic pathways, including PI3K-mTOR, SRC kinase, and the SHC-RAS-RAF-MEK-ERK cascade. Concurrently, ADAM10-mediated cleavage sheds s-NLGN3 from glioma cell membranes, establishing autocrine positive feedback.

In addition to neuronal regulation of synaptic transmission, Glioma cells appear to have also learned to respond to neuronal signals by the paracrine signaling of neural plasticity. Neurexins (NRXNs) and Neuroligins (NLGNs) are synaptic cell adhesion molecules that mediate presynaptic-postsynaptic neuronal connections and play critical roles in synapse development and signaling (54, 55). Neuroligin-3 (NLGN3) is predominantly distributed within the postsynaptic membranes of neurons and OPCs (56). This protein is released from these membranes in an activity-dependent manner, with secreted NLGN3(s-NLGN3) being cleaved from neurons and oligodendrocyte progenitor cells (OPCs) by A Disintegrin And Metalloproteinase10 (ADAM10) (57). As postsynaptic regulators of synaptic plasticity (58), s-NLGN3 critically mediates neuromodulation in gliomas by binding to glioma cell membranes (59). Gαi1/3 is activated by s-NLGN3 induction and mediates downstream oncogenic signaling pathways (60). Additionally, s-NLGN3 activates the PI3K-mTOR pathway, thereby promoting the proliferation and migration of glioma cells (61, 62). Unexpectedly, P13K upregulates NLGN-3 gene expression in glioma cells, generating more sNLGN-3 in the glioma microenvironment (57, 61) (**Figure 4**).

ADAM10 is highly enriched in synaptic vesicles (63). Reportedly, treatment with ADAM10 inhibitors suppresses the growth of adult and pediatric glioblastoma cells, an effect mediated by blocking ADAM10-dependent release of NLGN3 from neurons (64). s-NLGN3 shedding in the tumor microenvironment also drives optic pathway glioma (OPG) formation and growth. Mutations in the tumor suppressor gene *NF1* (neurofibromatosis 1) in retinal neurons and increased optic nerve activity were both associated with increased NLGN3 shedding (43). Notably, NLGN3 is not the exclusive regulator of activity-dependent glioma growth, as NLGN3 deficiency only partially attenuates glioma cell mitogenic potential rather than completely abolishing it.

### 3.3 BDNF

Brain-derived neurotrophic factor (BDNF) is a survival factor for certain neurons during development (65). Signals via two different types of receptors: myosin-related kinase (Trk) B and p75 kilodalton neurotrophic receptor (p75). BDNF exerts divergent roles in distinct cell types and microenvironments, potentially exhibiting either oncogenic or tumor-suppressive effects (66). Overexpression of BDNF and/or Trk-B has been reported in multiple cancer types (67). However, in the healthy brain, BDNF functions as a paracrine trophic factor to promote adaptive synaptic plasticity (68). During cognitive activities like thinking and learning, neuronal activity orchestrates BDNF gene transcription, mRNA trafficking to dendrites, and BDNF protein secretion (69–72). BDNF has been shown to promote proliferation and differentiation of NSCs—particularly into the oligodendroglial lineage—in a dose-dependent fashion, a process that promotes myelin remodeling and is regulated by insulin (73, 74). Interestingly, insulin can also promote the proliferation and survival of glioblastomas (75). BDNF regulates malignant synapse-like connections between neurons and glioma cells in malignant gliomas. BDNF signaling via the tropomyosin-related kinase B (Trk-B) receptor promotes trafficking of AMPA receptors to glioma cell membranes, thereby modulating the amplitude of postsynaptic currents (28). Consequently, neurons are potently driven to promote malignant tumor proliferation via synaptic-like connections.

### 3.4 Neural network remote regulation of glioma growth

Neuromodulation in gliomas is not regional, with gliomas forming a close connection with neural networks (76, 77). The magnitude of regulation of gliomas growing in different brain regions varies, which is influenced by the surrounding neural network environment and conduction. Research finds that gliomas are more frequent in cortical regions that inherently have higher activity levels (78). Furthermore, the type of neurons that form synaptic connections with gliomas varies depending on the location in the brain region, with mostly long-distance glutamatergic neurons in the cortex and short-distance GABAergic neurons in the striatum (79). This is similar to the synaptic connections between OPC and neurons (80).

Gliomas have been shown to integrate into the brain’s network structure, which encompasses connections among tumor-tumor, neuron-tumor, and tumor-other cell-type interactions (81). In the network structure, cancer cells are interlinked through specialized membranous conduits, called tumor microtubules (TM) (76). However, this interconnection is not ubiquitous. The cellular network formed by TMs contributes to an enhancement in the stemness characteristics and drug resistance of the tumor (82). Based on the network structure, neuronal projections from brain regions remote from the primary tumor contribute to tumorigenesis. Activation of neurons contralateral to gliomas using chemical genetics was revealed to promote not only glioma proliferation but also early infiltration. Surprisingly, severing the interhemispheric connections inhibits the activity-dependent acceleration of infiltration observed in intact controls, while mechanistic investigations identify Semaphorin 4F (SEMA4F) as a key mediator linking remote neuronal activity to glioma progression (83). SEMA4F is expressed by neurons and OPCs, and it stimulates OPC differentiation (84). In the migration of OPCs, Sema4F contributes to the correct migration of OPCs along the nerve, thereby preventing cell dispersion and intermingling (85). Diffuse midline glioma (DMG), a malignant pediatric tumor originating in the midline of the brain (86), arises from and closely resembles oligodendroglial lineage precursors regardless of its specific anatomical location (23). Recent studies reveal that mesencephalic cholinergic nuclei drive proliferation of both healthy OPCs and DMG cells in their projection targets via a circuit-dependent mechanism, providing the first evidence for distance regulation by cholinergic neuronal activity (87).

This phenomenon highlights the requirement for global neural activity in glioma development, which is strongly correlated with the migration of OPCs during repair and remodeling processes. Disruption of this neural pathway could potentially impede glioma progression.

## 4 Neural signals influencing glioma migration

It is well established that the dissemination of glioma cells contributes to their incurability (88), yet this process differs fundamentally from the metastasis of other solid cancers, which typically do not spread to distant organs (89). There are several possible reasons: First, although glioma cells bind to blood vessels, they may not be able to break through the basement membrane into the vasculature system (90). Second, extra-neural tissues may lack an appropriate growth microenvironment to support glioma proliferation. Clinical investigations have shown that following complete resection, postoperative recurrence predominantly occurs in the local white matter (91). The neuronal soma resides in the gray matter, while axons—including those forming the corpus callosum, the largest interhemispheric commissure—occupy the white matter, which is composed of a broad array of neural fibers (92, 93). Gliomas that spread along the white matter bundles of the corpus callosum are called butterfly gliomas (94). The prognosis for these patients is often poor, and severing the corpus callosum can largely prevent the spread of gliomas (83). The axonal architecture in the white matter creates a more permissive microenvironment for the dissemination of glioma cells.

White matter, situated beneath the gray matter cortex, consists of myelinated neuronal fibers that facilitate rapid signal transmission within the brain (95). Myelin plays a critical role in tumor spread. It serves as a highly permissive substrate for glioma cell adhesion and migration (96). The microenvironment of the CNS inherently exhibits resistance to glioma cell infiltration. Inhibitory molecules in CNS myelin (e.g., Nogo/Semaphorins/Ephrins, etc.) also suppress glioma cell migration and proliferation (97). This is based on another crucial function of myelin: to prevent excessive axonal regeneration, sprouting, and cellular infiltration into the brain parenchyma (98). Neuronal activity induces adaptive changes in myelin structure and function. Correspondingly, this activity significantly influences the invasive behavior of glioblastoma cells, including the formation, growth, and movement of TMs (99). Neurons paradoxically exhibit tumor-promoting effects on gliomas, though emerging studies reveal that adult post-mitotic neurons can induce apoptosis in both murine and human glioma cells (100). Moreover, *in vitro* co-culture reveals that the migratory ability of glioblastoma cells is inhibited by contact with neurons (101). The underlying mechanism may involve neuron-regulated glioma cells exhibiting characteristics resembling those of OPCs (8). Overall, neurons normally regulate OPCs to promote myelination repair, but may pathologically facilitate glioma migration along axons by misidentifying tumor cells as OPCs.

## 5 Neural networks enhance glioma cell stemness

The cellular composition of glioma is not homogeneous (102). Glioma cells with stemness, called glioma stem cells (GSCs), promote heterogeneity and drug resistance in gliomas (103). As normal stem and progenitor cells participate in tissue development and repair, these developmental programs re-emerge in CSCs to support the development and progressive growth of glioma (104). Current research suggests that GSC may be derived from NSCs residing in the SVZ in adults, as they share many common features (105, 106). The researchers believe that if GSCs are the glioma cells responsible for generating the tumors, then the developmentally analogous relationship is the NSC-OPC axis (107). In fully developed individuals, NSCs can differentiate into OPCs as the primary source of myelination contribution (108). Olig2 is highly expressed in OPCs as well as in GSCs (109). Culturing glioma cells with conditioned medium from OPCs, which contains secreted factors, indicates that soluble factors secreted by OPCs enhance the stem-like properties of glioma cells, thereby contributing to tumorigenesis, therapeutic resistance, and recurrence (110). OPCs and macrophages/microglia form a distinct microenvironment for glioma cells at the tumor boundary, with particularly prominent aggregation in recurrent lesions. In this microenvironment, OPCs may drive the acquisition of stemness in glioma cells (91, 110, 111). Neuronal activity enhances the stemness of glioma cells. Studies have shown that exosomes derived from active neurons promote glioma progression and radioresistance by inducing phenotypic and metabolic transformation of GSCs (112). In summary, we suggest that the aggregation of OPCs at the tumor may misregulate the enhancement of glioma stemness, and this regulation can be potentiated by electrical activity stimulation.

## 6 Neural-immune interplay in glioma

In addition to directly mediating tumor growth, neurons can promote the tumor by modulating immune cell function. Astrocytes perform supporting functions for neurons and oligodendrocytes (113). Microglia are recognized as mononuclear phagocytic cells that play a significant role in immune response and homeostasis within the CNS (114). They contribute to the formation, maintenance, and reshaping of neuronal circuits by clearing dead cells and participating in neural repair through pruning (115, 116). In neuroplasticity, complex interactions between neurons, T cells, and microglia (117). Neurons play a crucial role in regulating microglia activation, as neurons secrete factors such as CD200 (118), SEMA3A (119), and CX3CL1 (120) can modulate microglial cell properties to different degrees. Whereas this regulation promotes the process of neuronal repair and remodeling in the normal brain, in the glioma setting, neurons produce reduced mid-term to activate T cells, which in turn leads to an increase in T cell Ccl4 secretion and microglial cell secretion of Ccl5 to sustain glioma cell growth (117).

Overall, while glioma growth stimulates immune cell repair and participates in neural remodeling, signaling impulses from neurons can, in turn, facilitate this process. However, this process seems to be exploited by the glioma cells for the use of self-growth.

## 7 Disturbance of neural network by glioma

Neuron–glioma interactions are bidirectional. Based on subdural electrocorticography, sampling of normal and glioma-infiltrated cortex during speech showed that glioma infiltration affected the brain’s ability to encode information during nuanced tasks (121). Recent studies have revealed that tumor-associated cortical networks exhibit hyperexcitability (8). Tumor-induced disruption of synaptic network activity in the peritumoral region leads to alterations in network excitability (122). Although neuronal over-excitation maintains task-specific neuronal responses, the tumor-affected cerebral cortex loses the ability to decode complex words (123).

In addition to impairing brain function, epilepsy is diagnosed in 70–90% of patients with glioma (124). However, further investigation has revealed that the abnormal enhancement of peritumoral neuronal network activity and the prevalent epileptiform activity were closely associated with the formation of new synapses, with glioma cells forming these new synapses remaining as OPC-like cells (125). In the glioma-surrounding tissue, extracellular glutamate levels were found to be 100 times higher than in the unaffected brain (126). Glutamate secretion from gliomas stimulates peritumoral neuronal receptors, leading to neuronal hyperexcitability and epileptic seizures (127).

## 8 Conclusion and future directions

There is growing evidence that different types of cancers originate from distinct “progenitor cells”, which undergo the first or multiple genetic hits leading to the onset of cancer (128). Therefore, the origin and progression of cancer cells in different locations depend on the surrounding environment and cell type. Gliomas, the most prevalent primary malignant tumors in the adult CNS, are likely triggered by the daily remodeling and repair processes of glial cells, during which multiple factors induce malignant changes. OPCs, the most active stem cells in the brain and responsible for myelin plasticity, are also found aggregating around gliomas.

The underlying mechanism of this OPC aggregation—whether driven by reparative recruitment or malignant transformation during the initial repair process—remains inconclusive. Mosaic Analysis with Double Markers (MADM)-based lineage tracing revealed significant abnormal growth prior to malignancy only in OPCs (129). Notably, accumulating evidence has established that OPC aggregation significantly accelerates glioma progression. Stem cells not only have the mission of proliferation and differentiation but also require multiple factors (e.g., neuroregulatory signals, paracrine factors) to promote or inhibit the function (130). Similarly, in gliomas, the factors that regulate OPC also regulate the glioma cells and even form similar synaptic connections. It seems that brain cancer learns the mechanisms of neural plasticity.

However, these regulators also exhibit bidirectionality. As a key modulator of synaptic plasticity (72), BDNF contributes to physiological synaptic regulation through neuronal activity and drives tumor progression through BDNF-TrkB-mediated malignant synapse enhancement (28). Its effects are not unilaterally protumorigenic: mature BDNF/TrkB signaling drives glioma growth, migration, and anti-apoptotic effects, while proBDNF/p75NTR activation inhibits these processes (131). Additionally, lncRNA BDNF-AS suppresses malignancy by targeting RAX2 (132). This functional difference depends on the type of cells involved, the selective binding of receptor subtypes, and microenvironmental characteristics (66). GABA shows more pronounced bidirectionality (133). In DMG, NKCC1-mediated high intracellular Cl^-^ converts the action of GABA to membrane depolarization, promoting proliferation (51). Additionally, GABA maintains GSC quiescence for post-surgical recurrence (134). Conversely, GABA_a_R activation inhibits proliferation in low-grade gliomas via enhanced inhibitory signaling, although a mechanism potentially weakened by GABA_a_R downregulation in glioblastoma (135). *In vitro* experiment, neuronal GABA_a_R activation directly suppresses glioma growth (136). This bidirectionality resembles the functional differences of GABAergic signaling in OPC regulation (47). This suggests that targeted modulation of Cl^-^ currents in glioma cells may provide a novel therapeutic approach to halt tumor progression (137).

Current experimental models of tumor-neuron interactions predominantly rely on *in vitro* cell co-culture (138) or xenografts in immunodeficient mice (8, 61). Though they partially reflect the interactions between gliomas and neurons, they cannot replicate the 3D structure of *in vivo* neural circuits, neurotransmitter microenvironment, or brain region-specific neuroplasticity. However, related studies have made progress. An *in vitro* 3D model constructed using 3D bioprinting technology, consisting of an outer hemisphere containing neurons and an inner hemisphere containing glioma cells (139). Modeling glioblastoma invasion using human brain organoids (140). Co-culture system using patient-derived GBM organoids and human induced pluripotent stem cells (hiPSCs) (141). Despite these advancements, further optimization is still needed to more realistically simulate the physiological environment of tumor-neuron interactions *in vivo*. Another important consideration is that there are significant differences between pediatric and adult gliomas in terms of genetic background, site of origin, and clinical behavior (142). Adult gliomas often originate in the supratentorial region and are often accompanied by neuroplastic compensatory mechanisms. In contrast, pediatric gliomas predominantly occur in brain regions with active neurogenesis, including the brainstem and thalamus. The progression may be more closely linked to the active neuroplasticity during the brain development stage (143). Therefore, it is necessary to study the differences between the contributions of “developmental neuroplasticity” and “pathological neuroplasticity” in childhood and adult gliomas.

Molecules related to neuroplasticity may serve as potential targets for glioma treatment, but the specific mechanisms remain unclear. ADAM10 is highly expressed in gliomas; however, the mechanism by which ADAM10 balances neuroplasticity and glioma phenotypes through cleaving different substrates remains elusive (64). AMPAR is the core subtype of glutamate receptors. Pharmacological inhibition of AMPAR activity using Talampanel has demonstrated potential in the clinical management of newly diagnosed glioblastoma (144), but the impact of long-term AMPAR inhibition on normal neurological function has not yet been systematically validated. Cav3, as a T-type calcium channel in synaptic plasticity, can be utilized in inhibiting glioma development through disconnecting nerve cell and OPC-like glioma cell interaction (145, 146). Rabies-mediated genetic ablation of neurons halts glioblastoma progression (44). Unexpectedly, some commonly used drugs have been found to have tumor-promoting effects, such as Lorazepam (51). Currently, there is growing evidence that multiple neuroplasticity signals are exploited to influence the progression of gliomas, suggesting that learning and remodeling are closely related to the initiation and progression of gliomas. We believe that broader plasticity regulatory mechanisms can inspire the study of abnormal tumor proliferation. Meanwhile, based on the study and modulation of neuroplasticity, more effective treatments for controlling the progression of gliomas will be discovered.
